# Population‐level analysis of natural control of HIV infection in Zambia and South Africa: HPTN 071 (PopART)

**DOI:** 10.1002/jia2.26179

**Published:** 2023-10-27

**Authors:** Wendy Grant‐McAuley, Estelle Piwowar‐Manning, William Clarke, Autumn Breaud, Kidist Belay Zewdie, Ayana Moore, Helen Mary Ayles, Barry Kosloff, Kwame Shanaube, Peter Bock, Sue‐Ann Meehan, Gerald Maarman, Sarah Fidler, Richard Hayes, Deborah Donnell, Susan H. Eshleman, for the HPTN 071 (PopART) Study Team

**Affiliations:** ^1^ Department of Pathology Johns Hopkins University School of Medicine Baltimore Maryland USA; ^2^ Department of Epidemiology University of Washington Seattle Washington USA; ^3^ FHI 360 Durham North Carolina USA; ^4^ Zambart University of Zambia School of Public Health Lusaka Zambia; ^5^ Clinical Research Department London School of Hygiene and Tropical Medicine London UK; ^6^ Desmond Tutu TB Center Department of Paediatrics and Child Health Stellenbosch University Western Cape South Africa; ^7^ Centre for Cardio‐Metabolic Research in Africa Division of Medical Physiology Faculty of Medicine and Health Sciences Stellenbosch University Western Cape South Africa; ^8^ Department of Infectious Disease Imperial College London London UK; ^9^ Department of Infectious Disease Epidemiology London School of Hygiene and Tropical Medicine London UK; ^10^ Fred Hutchinson Cancer Research Center Seattle Washington USA

**Keywords:** HIV, controller, population, HPTN 071, Zambia, South Africa

## Abstract

**Introduction:**

HIV controllers have low viral loads (VL) without antiretroviral treatment (ART). We evaluated viraemic control in a community‐randomized trial conducted in Zambia and South Africa that evaluated the impact of a combination prevention intervention on HIV incidence (HPTN 071 [PopART]; 2013–2018).

**Methods:**

VL and antiretroviral (ARV) drug testing were performed using plasma samples collected 2 years after enrolment for 4072 participants who were HIV positive at the start of the study intervention. ARV drug use was assessed using a qualitative laboratory assay that detects 22 ARV drugs in five drug classes. Participants were classified as non‐controllers if they had a VL ≥2000 copies/ml with no ARV drugs detected at this visit. Additional VL and ARV drug testing was performed at a second annual study visit to confirm controller status. Participants were classified as controllers if they had VLs <2000 with no ARV drugs detected at both visits. Non‐controllers who had ARV drugs detected at either visit were excluded from the analysis to minimize potential confounders associated with ARV drug access and uptake.

**Results:**

The final cohort included 126 viraemic controllers and 766 non‐controllers who had no ARV drugs detected. The prevalence of controllers among the 4072 persons assessed was 3.1% (95% confidence interval [CI]: 2.6%, 3.6%). This should be considered a minimum estimate, since high rates of ARV drug use in the parent study limited the ability to identify controllers. Among the 892 participants in the final cohort, controller status was associated with biological sex (female > male, *p* = 0.027). There was no significant association between controller status and age, study country or herpes simplex virus type 2 (HSV‐2) status at study enrolment.

**Conclusions:**

To our knowledge, this report presents the first large‐scale, population‐level study evaluating the prevalence of viraemic control and associated factors in Africa. A key advantage of this study was that a biomedical assessment was used to assess ARV drug use (vs. self‐reported data). This study identified a large cohort of HIV controllers and non‐controllers not taking ARV drugs, providing a unique repository of longitudinal samples for additional research. This cohort may be useful for further studies investigating the mechanisms of virologic control.

## 1 INTRODUCTION

HIV controllers naturally suppress their HIV viral load (VL) to low levels without antiretroviral treatment (ART) [1]. Elite controllers maintain VLs <50 copies/ml, while viraemic controllers maintain VLs <2000 copies/ml [2, 3, 4]. HIV control is associated with reduced morbidity and mortality [3, 5], consistent with the correlation between VL set point and HIV disease progression [6, 7, 8, 9, 10, 11, 12, 13]. Control develops early in infection [3, 14, 15]. In the absence of antiretroviral treatment (ART), controllers often experience an extended period with normal CD4^+^ T cell counts (up to 25+ years [1]) before the immune system decline and progression to AIDS [3, 5, 16].

Previous population‐level studies have reported the prevalence of elite and viraemic controllers as <1% [3, 17, 18, 19] and <4% [3, 20], respectively. It can be difficult to compare data across studies due to differences in the criteria used to define controller status (e.g. VL cut‐off and duration of viral suppression) and methods used to assess antiretroviral (ARV) drug use (e.g. drug testing and self‐report). Few studies have evaluated demographic factors associated with HIV control. A natural history study from the United States Department of Defense evaluated a cohort of 4586 military personnel that included 25 elite controllers and 153 viraemic controllers; ART status was based on self‐report with medical records review. In that study, viraemic control was more common in persons with African American ethnicity (vs. European American ethnicity); there was no association of elite or viraemic control with transmission route (e.g. sexual and injection drug use) or demographic variables (e.g. age and biological sex) [3].

In this report, we evaluated factors associated with viraemic control in a community‐randomized trial conducted in Zambia and South Africa: HIV Prevention Trials Network (HPTN) 071 (PopART). This trial evaluated the impact of a combination prevention strategy, including universal testing and treatment, on HIV incidence [21]. Communities were randomized into three study arms (Arm A: intervention package with ART provided at any CD4 count, Arm B: intervention package with ART provided according to local treatment guidelines and Arm C: standard‐of‐care). Local guidelines for ART initiation were different in the two study countries and changed over the course of the trial; by the end of the trial, universal ART was available to all participants diagnosed HIV positive [21, 22]. The impact of the intervention was evaluated in a population cohort (PC) of >48,000 participants who were followed for up to 3 years with annual home visits that included HIV testing and sample storage. VL and ARV drug testing were performed retrospectively for all HIV‐positive participants 2 years after the start of the study intervention [21, 23]. At that point in the trial, >70% of study participants were using ARV drugs, and >90% of those participants had VLs <400 copies/ml [23]. ART use was associated with older age, female sex, enrolment year, seroconverter status and self‐reported ART [23].

We identified viraemic controllers and non‐controllers in the HPTN 071 cohort and examined demographic factors associated with HIV control. All participants assessed in this report had prevalent HIV infection (duration >2 years). By definition, viraemic controllers were virally suppressed in the absence of ART. To minimize confounding by factors associated with ART in the HPTN 071 trial, this report only included non‐controllers who had no ARV drugs detected.

## 2 METHODS

### 2.1 Study cohort

This research was conducted using samples and data collected in the HPTN 071 (PopART) trial (NCT019000977; 2013–2018) [21]. Samples were collected at annual study visits referred to as PC0, PC12, PC24 and PC36 [21]. This report included 4101 participants who were HIV positive at the start of the study intervention (PC0) and had a sample collected 2 years after enrolment (PC24). Participants with acute HIV infection at enrolment were excluded from the analysis.

### 2.2 Laboratory methods

Herpes simplex virus type 2 (HSV‐2) testing was performed at study sites using the Kalon HSV2 immunoglobulin G enzyme‐linked immunosorbent assay (ELISA; Kalon Biological), as described [24]. VL and ARV drug testing were performed at the HPTN Laboratory Center (Johns Hopkins University). VL testing was performed using the RealTime HIV‐1 Viral Load assay (Abbott Molecular; validated dilution method; limit of quantitation: 400 copies/ml). VLs were set at 399 copies/ml for samples with no RNA detected or RNA detected <400 copies/ml. ARV drug use was assessed using a qualitative assay that detects 22 ARV drugs in five drug classes (limit of detection: 2 or 20 ng/ml, depending on the drug) [25]. VL and ARV drug testing were performed previously for all HIV‐positive study participants at the PC24 visit [21, 23]. Samples from a second visit (PC0, PC12 or PC36) were selected for additional testing for participants who had VLs <2000 copies/ml with no ARV drugs detected at the PC24 visit. The visit selected for further testing was based on the availability of existing data and/or samples; if participants had samples available from multiple visits, the sample collected earlier in the study was selected for testing.

### 2.3 Statistical methods

Participants classified as viraemic controllers had VLs <2000 copies/ml with no ARV drugs detected at two annual study visits. This classification approach is consistent with methods described in previous reports [2, 3, 4, 26]. Univariate analyses were performed using the *t*‐test for continuous variables and the chi‐square test (without correction) for categorical variables. Statistical analyses were performed using R v4.1.2 [27].

### 2.4 Informed consent

Study participants provided written informed consent prior to enrolment in HPTN 071, which was approved by the institutional review board and ethics committees at the London School of Hygiene and Tropical Medicine, the University of Zambia and Stellenbosch University.

## 3 RESULTS

**Figure 1:**
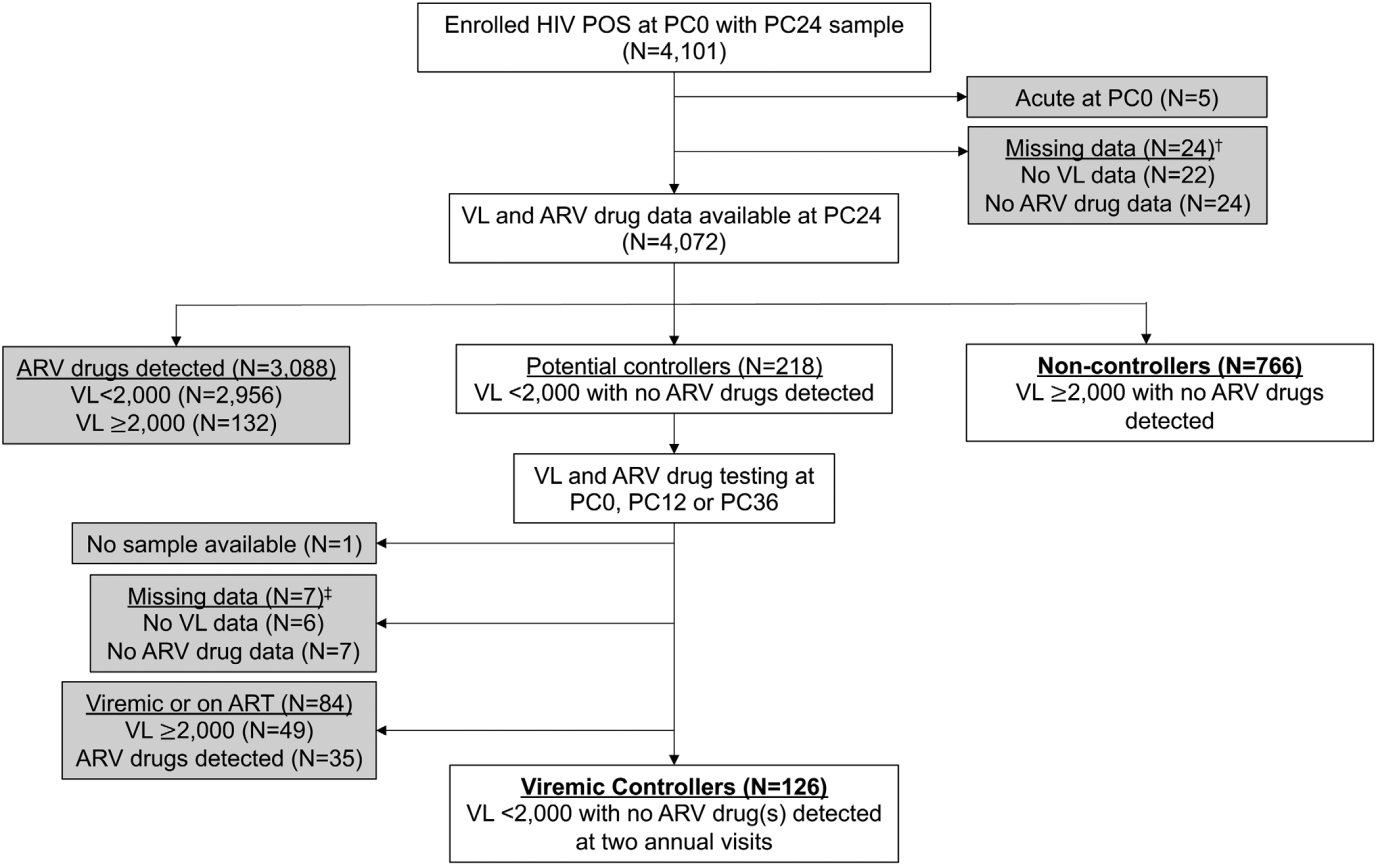
Identification of viraemic controllers and non‐controllers in HPTN 071 (PopART). The figure shows the approach used to identify viraemic controllers and non‐controllers. The cohort included HPTN 071 (PopART) participants who were HIV positive at study enrolment (PC0) and had a sample available from a study visit 2 years later (PC24). Viral load and antiretroviral (ARV) drug data obtained at the PC24 visit were used to identify potential controllers and non‐controllers. Participants who had viral loads ≥2000 copies/ml with no ARV drugs detected at PC24 were classified as non‐controllers. Participants who had viral loads <2000 copies/ml with no ARV drugs detected at PC24 were classified as potential controllers. Testing was performed at a second study visit (PC0, PC12 or PC36) to identify the subset of potential controllers who met the criteria for viraemic control (viral load <2000 copies/ml with no ARV drugs detected at two visits at least 1 year apart). Shaded boxes indicate participants who had undetermined controller status or were excluded from the analysis due to ARV drug use. ^†^ Twenty‐two of the 24 participants in this group did not have viral load or ARV drug data from PC24; the other two participants in this group did not have ARV drug data from this visit. ^‡^ Six of the seven participants in this group did not have viral load or ARV drug data from a second visit; in the other case, the participant did not have ARV drug data from this visit. Abbreviations: ARV, antiretroviral; *N*, number; PC, population cohort; PC0, study visit at the start of the study intervention; PC12, study visit 12 months after the start of the study intervention; PC24, study visit 24 months after the start of the study intervention; PC36, study visit 36 months after the start of the study intervention; POS, positive; VL, viral load.

HPTN 071 included 4101 participants who were HIV positive at the start of the study intervention (PC0) and had a sample stored 2 years later (PC24; Figure 1); five participants who had an acute infection at enrolment were excluded from the analysis. VL and ARV drug testing were performed previously using samples collected at the PC24 visit [21, 23]; VL and ARV data were obtained for 4072 (99.3%) of the 4101 participants. ARV drugs were detected in samples from 3088 (75.8%) of those participants; those participants were excluded from analysis to minimize potential confounders associated with ART access and uptake. The remaining 984 participants included 766 participants with VLs ≥2000 copies/ml who were classified as non‐controllers and 218 participants with VLs <2000 copies/ml who were classified as potential controllers.

To determine which of the 218 potential controllers met the criteria for viraemic control, VL and ARV drug testing were performed using samples collected from a second visit (1–2 years earlier or 1 year later); results were obtained for 210 (96.3%) of the 218 potential controllers. Based on those results, 126 potential controllers were classified as viraemic controllers (VL <2000 copies/ml with no ARV drugs detected at two visits at least a year apart). The remaining 92 participants were excluded from further analysis, since controller status could not be determined based on available data. This group included eight participants who had no additional samples or were missing VL and/or ARV drug data from an additional visit, 49 participants who had VLs ≥2000 copies/ml at the second visit tested; and 35 participants who had VLs <2000 copies/ml with ARV drugs detected at the second visit tested. The final cohort included 126 controllers and 766 non‐controllers. The prevalence of viraemic control among the 4072 participants with PC24 data was 3.1% (95% confidence interval [CI]: 2.6%, 3.6%).

We compared demographic and other characteristics for the 126 controllers and 766 non‐controllers (Table 1). Viraemic control was associated with biological sex (higher among women, *p* = 0.027). There was no significant association between viraemic control and age, study country or HSV‐2 status at PC0.

**Table 1:** Factors associated with viraemic control in HPTN 071 (PopART)

	Total (*n* = 892)	Controllers^a^ (*n* = 126)	Non‐controllers^b^ (*n* = 766)	*p*‐Value
**Viral load (copies/ml)** ^c^				N/D^d^
Mean (IQR)	86,276 (399, 72,358)	700 (399, 973)	100,353 (8,635, 89,160)	
**Sex**				**0.027**
Female	722	111 (15.4%)	611 (84.6%)	
Male	170	15 (8.8%)	155 (91.2%)	
**Age (yrs)** ^e^				0.47
Mean (SD)	31.8 (7.0)	31.5 (6.5)	31.9 (7.0)	
**Age (five‐category)** ^e^				0.22
18–24	158	22 (13.9%)	136 (86.1%)	
25–29	213	27 (12.7%)	186 (87.3%)	
30–34	210	38 (18.1%)	172 (81.9%)	
35–39	157	24 (15.3%)	133 (84.7%)	
40+	154	15 (9.7%)	139 (90.3%)	
**Country**				0.15
South Africa	443	70 (15.8%)	373 (84.2%)	
Zambia	449	56 (12.5%)	393 (87.5%)	
**HSV‐2 status** ^f^				0.29
Positive	704	103 (14.6%)	601 (85.4%)	
Negative	167	18 (10.8%)	149 (89.2%)	
Indeterminate	8	2 (25%)	6 (75%)	

*Note*: The table shows factors associated with viraemic control. Statistically significant values are shown in bold text.

Abbreviations: ARV, antiretroviral; HSV‐2, herpes simplex virus type 2; IQR, interquartile range; N/D, not determined; SD, standard deviation; yrs, years.

^a^ Participants were classified as controllers if they had viral loads <2000 copies/ml with no ARV drugs detected at two annual study visits.

^b^ Participants were classified as non‐controllers if they had a viral load ≥2000 copies/ml with no ARV drugs detected at the PC24 visit.

^c^ Data from viral load testing are shown for the PC24 visit.

^d^ *p*‐Value for viral load was not reported because this factor was used to identify controllers.

^e^ Age was analysed as a continuous variable (years) and by age category (18–24 years, 25–29 years, 30–34 years, 35–39 years and 40+ years).

^f^ Data for HSV‐2 status are shown for the PC0 visit; data were not available for 13 participants (3 controllers and 10 non‐controllers).

## 4 DISCUSSION

To our knowledge, this report presents the first large‐scale, multisite, population‐level study evaluating the prevalence of viraemic control and associated factors in Africa. The study was conducted in a generalized epidemic setting in two sub‐Saharan African countries and included men and women from urban and peri‐urban communities. Using the controller definition described in this report, we identified 126 viraemic controllers and 766 non‐controllers who were not on ART. An advantage of this study was that a biomedical assessment was used to assess ARV drug use. This assay detects 22 drugs in five classes, including those recommended for ART in the study countries at the time the trial was performed. Previous studies have demonstrated that self‐report of ART can be unreliable, as some participants in clinical trials and research studies may not accurately report their ART status [28, 29, 30]. Some persons with HIV may also be taking ARV drugs for HIV pre‐exposure prophylaxis (if they are unaware of their HIV status), post‐exposure prophylaxis or other reasons (e.g. recreational use [31]) that could impact HIV VL; these data may not be included in questionnaires or captured in self‐reported data sets.

The prevalence of viraemic controllers identified in this report (3.1%) is similar to results from population‐level studies conducted in the United Kingdom (2.7%) [20] and the United States (3.3%) [3]. This should be considered a minimum estimate, since we could not determine controller status for all HPTN 071 participants, largely due to high rates of ARV drug use. Over 75% of participants were taking ARV drugs at the PC24 visit, and 35 (16.1%) of the 218 potential controllers (those who had VLs <2000 copies/ml with no ARV drugs detected at the PC24 visit) were taking ARV drugs at another visit. Some of these participants may have been classified as controllers if they were not taking ARV drugs.

In the Department of Defense study noted above, viraemic control was not associated with biological sex [3]. However, different results were obtained in a subsequent report that evaluated a subset of that cohort (hospitalized participants) and compared two participant subgroups: controllers (elite and viraemic controllers combined) versus non‐controllers who were not on ART; in that analysis, HIV control was more common in women [32]. Those results are consistent with our finding of a higher frequency of viraemic control in women and with a prior report that found lower VLs in women compared to men in the absence of ART [9].

In the HPTN 071 cohort, viraemic control was more common in South Africa than in Zambia, although these findings were not statistically significant. Previous studies found above‐average rates of HIV control in South Africa, ranging from 5.6% in a cohort of young women [33] to 17.2% in a cohort from a single township that assessed ARV drug use at one visit using self‐reported data [34]. Local differences in host or viral genetics may be responsible for regional differences in rates of viraemic control. The prevalence of viraemic control may increase over time in regions with established HIV epidemics, since HIV control is associated with improved health outcomes and increased life expectancy [3, 5, 16]. We did not observe an association of viraemic control with HSV‐2 seropositivity, age or other factors analysed.

This study had several limitations. First, high rates of ARV drug use limited our ability to identify controllers. This study used VL and ARV drug data that were obtained in a prior study 2 years after the start of the study intervention. More participants may have met the criteria for viraemic control if testing had been performed at the study entry. For this reason, our estimated prevalence of 3.1% should be considered a minimum estimate. Second, HPTN 071 included a disproportionate number of women and was conducted in Zambia and South Africa, where almost all infections are caused by HIV subtype C. In a prior study, we performed HIV genotyping and subtyping for a subset of 758 participants in HPTN 071; 95.9% of those participants had subtype C HIV infection [35]. These factors may limit the generalizability of our findings to other cohorts and to regions where other subtypes predominate. Third, the limit of quantification of the assay used for VL testing was not low enough to differentiate between elite and viraemic controllers; therefore, we were unable to evaluate factors associated with elite HIV control. Fourth, the assay used for ARV drug testing only detects recent ARV drug use; participants who had low VLs with suboptimal ART adherence may have been misidentified as controllers. Fifth, VL and ARV testing were only performed at two annual study visits; a longer duration of drug‐free viraemic control was not evaluated. Sixth, HIV transmission was not evaluated due to low VLs for most controllers, the lack of enrolled sexual partners and limitations in participant consent for phylogenetic analysis. Seventh, most controllers in this study had very low HIV VLs, which limited our ability to evaluate the association of virologic factors with HIV control. Finally, the HPTN 071 study did not include the collection of CD4 count data, consent for DNA analysis or storage of cellular samples. Therefore, it was not possible to evaluate the association between controller status and CD4 cell count, human leukocyte antigen (HLA) type or other factors related to cellular immunity. We are now extending these studies to evaluate the association of serologic factors and HIV control.

## 5 CONCLUSIONS

The prevalence of viraemic control in this population‐based study was 3.1%. Viraemic control was associated with biological sex (women > men). This study identified a large cohort of controllers and non‐controllers. Further characterization of this cohort may provide insights into the mechanisms of HIV control.

## COMPETING INTERESTS

None of the authors has competing interests or potential competing interests.

## AUTHORS’ CONTRIBUTIONS

WG‐M: designed the study, performed laboratory testing, analysed data and drafted the manuscript; EP‐M: HPTN 071 LC QA/QC Representative; WC: responsible for antiretroviral drug testing; AB: performed antiretroviral drug testing; KBZ: assisted with data management; AM: HPTN 071 Study Coordinator; HMA: HPTN 071 Zambia Site PI; BK: provided laboratory support for HPTN 071 in Zambia; KS: HPTN 071 (PopART) Zambia Site Investigator; PB: HPTN 071 South African Site Co‐PI; SAM: HPTN 071 Research Manager in South Africa; GM: provided laboratory support for HPTN 071 in South Africa; SF: HPTN 071 Protocol Co‐Chair; RH: HPTN 071 Protocol Chair; DD: HPTN 071 Statistician; SHE: HPTN 071 Virologist, designed the study, analysed data and drafted the manuscript. All authors contributed to the manuscript and approved the submitted version.

## FUNDING

This work was supported by the National Institute of Allergy and Infectious Diseases (NIAID) of the National Institutes of Health (NIH) through R01‐AI095068, the National Institute of General Medical Sciences (NIGMS) through R01‐GM136724 and by the HIV Prevention Trials Network (HPTN), which is sponsored by the NIAID under Cooperative Agreements UM1‐AI068619, UM1‐AI068617 and UM1‐AI068613, with funding from the U.S. President's Emergency Plan for AIDS Relief (PEPFAR). Additional support was provided by the Division of Intramural Research, NIAID. Additional funding for the HPTN 071 (PopART) trial was provided by the International Initiative for Impact Evaluation (3ie) with support from the Bill & Melinda Gates Foundation, as well as by NIAID, the National Institute on Drug Abuse (NIDA) and the National Institute of Mental Health (NIMH), all part of NIH. RH also received support from the UK Medical Research Council (MRC) and the UK Department for International Development (DFID) under the MRC/DFID Concordat agreement, which is also part of the EDCTP2 programme supported by the European Union (MR/R010161/1).

## DISCLAIMER

The content is solely the responsibility of the authors and does not necessarily represent the official views of the NIAID, NIMH, NIDA, PEPFAR, 3ie or the Bill & Melinda Gates Foundation.

## Data Availability

The data sets used and/or analysed during the current study are available from the corresponding author on reasonable request.
